# Tranexamic acid in spinal surgery: a stratified protocol from the surgeon's point of view

**DOI:** 10.1016/j.bas.2026.105989

**Published:** 2026-02-23

**Authors:** D. Rodríguez, J. Melero, A. Pont, V. Calvet, G. Ruiz, L. Campana, M.C. Raya, C. Jiménez, E. Vila, U. Rodríguez, D. Bartolomé, A. Del Arco, G. Vilá, A. Isart, D. Manzano, J. Lafuente

**Affiliations:** a Spinal Surgery Unit, Hospital del Mar, Barcelona, Spain; b Universitat Pompeu Fabra, Barcelona, Spain; c Health Services Research Group, Hospital del Mar Research Institute, Barcelona, Spain, CIBER in Epidemiology and Public Health, CIBERESP, Madrid, Spain; d Neurosurgery Department, Hospital Churruca Visca, Buenos Aires, Argentina; e Haematology Department Hospital del Mar, Barcelona, Spain; f Anesthesiology Department Hospital del Mar, Barcelona, Spain

**Keywords:** Tranexamic acid, Spine surgery, Blood loss, Risk assessment

## Abstract

**Introduction:**

Intraoperative bleeding in spinal surgery remains a major concern, given its association with increased morbidity, prolonged hospitalization, and greater transfusion requirements. Tranexamic acid (TXA), a synthetic antifibrinolytic, has shown consistent efficacy in minimizing blood loss across diverse surgical contexts. Nevertheless, its application in spinal surgery must be individualized, taking into account bleeding risk, surgical complexity, and patient comorbidities.

**Research question:**

Following the creation of a dedicated Spine Unit, our objective was to design and validate a protocol for TXA administration and blood transfusion (BT) specific to spinal procedures.

**Materials and methods:**

We retrospectively reviewed 1223 spinal surgeries in 1059 patients aged over 13 years, conducted from April 2018 to April 2023. TXA use was guided by a stratified bleeding risk model incorporating surgical approach, use of minimally invasive spinal surgery (MISS), number of treated levels, instrumentation, reoperations, and diagnosis of complex spine surgery (CSS). Preoperative variables included hemoglobin level, anticoagulant/antiplatelet therapy, and coagulopathy. Outcomes assessed were transfusion needs, hemoglobin drop, estimated blood loss (EBL), complications, infections, length of stay, and mortality. Propensity scores (c-statistic = 0.756) and standardized morbidity ratio weighting addressed treatment selection bias.

**Results:**

TXA was significantly associated with non-MISS procedures involving instrumentation or high-bleeding-risk conditions such as metastatic tumours or deformities. EBL >500 mL increased the odds of TXA use 6.5-fold. TXA was not linked to thromboembolic or renal complications. Transfusion was associated with high-risk diagnoses, infections, prolonged aPTT, multilevel surgery, and EBL >500 mL, and predicted worse outcomes, including longer stays and higher mortality. TXA significantly reduced mortality (OR = 0.25).

**Discussion and conclusions:**

Our findings support a stratified TXA protocol incorporating patient- and procedure-related risk factors. Such an approach enhances perioperative safety, reduces transfusion requirements, and improves survival in high-risk spinal surgeries.

## Highlights

• Stratified protocol for tranexamic acid tailored to spinal surgery risk.
• TXA usage independently reduced mortality without added complications in our study population.
• The model integrates patient and procedure factors to guide TXA indication.
• TXA particularly beneficial in deformity, metastasis, and multilevel (≥3) cases.
• Protocol will enhance perioperative safety and reduce transfusion needs.

## 1 Introduction

### 1.1 Background

More than a thousand years after the first description in Cordoba (Spain) of a patient's death during a cervical spine surgery by Abul Qasim al-Zahrawi (Abulcasis) (López Piñero, 2002), intraoperative uncontrolled bleeding continues to be the most feared challenge to the anatomical knowledge, experience and skilfulness of the spinal surgeon. A myriad of perioperative (periop) tools have been tried and developed since then to minimize blood loss during spine surgery (Le et al., 2018; Mikhail et al., 2020), and among them the two main antifibrinolytic agents used in clinical practice: the tranexamic (TXA) and ε-aminocaproic (EACA) acids. Both compounds are lysine analogues that bind to and prevent activation of plasminogen, thereby inhibiting fibrinolysis and promoting clot stabilization (Wardrop et al., 2013). TXA is 7 to 10 times more potent than EACA and has proved to be effective with therapeutic Level of Evidence I when reducing the intra and postoperative (postop) estimated blood loss (EBL) and to a lesser degree the blood transfusion (BT) requirements during spine surgery as reported through several prospective, multicentric and randomised studies (Colomina et al., 2017; Goobie et al., 2018; Lei et al., 2022). TXA use in spinal surgery was mainly promoted from 2010 ahead, when blood saving techniques and allogeneic transfusions were enlarged with the preoperative (preop) haemoglobin (Hb) optimization, minimizing intraoperative (intraop) bleeding through antifibrinolytic drugs and improvement of patient's tolerance to anaemia concepts (Patient Blood Management) (see Table 3, Table 4, Table 5, Table 6).

Although the effectiveness of TXA in spine surgery is well documented, there is a lack of clinical trials about its safety related to thromboembolic events and renal complications during the periop period (Basora and Colomina, 2020). According to the Food and Drugs Administration (FDA) recommendations, it is contraindicated in patients with known allergy to TXA, intracranial bleeding, known defective color vision, history of venous or arterial thromboembolism, or active thromboembolic disease (Chauncey and Wieters, 2025). Its intravenous (IV) use in spinal surgery is considered off-label, and its more frequent dosage is 10-30 mg/kg in initial bolus followed by continuous perfusion of 1-2 mg/kg/h. Even topical use of TXA has been explored and systematic reviews suggest similar hemostatic efficacy compared with intravenous TXA (Winter et al., 2016).

### 1.2 Rationale and knowledge gap

The evidence about effectiveness of TXA reducing EBL and BT in spinal surgery is not so strong as in total hip or knee replacements, mainly because randomized control trials in spinal surgery include different primary diagnosis, surgical locations or levels of fusion, therefore with different bleeding risks. Recent meta-analyses (Qin et al., 2022; Lu et al., 2018) have pointed to the need of stratification of the individual bleeding risk (BR) in spinal surgery, according to the identification of those patients whom the antifibrinolytic therapy can be more beneficial. TXA administration in spinal surgery alos shifted the paradigm in transfusion, requiring a consensus among different professionals involved in patient's periop care including nurses, on-call doctors, Anesthesiology, Haematology and Rehabilitation teams (Basora and Colomina, 2020).

### 1.3 Objective

As a Spinal Unit was formally constituted in 2018 at our center by two Neurosurgeons and two Orthopaedic Surgeons (Rodríguez et al., 2023) it looked desirable and essential to develop a formal protocol for the use of TXA and BT in the spinal procedures performed in our center, which is the main objective of this study.

## 2 Material and methods

**Fig. 1:**
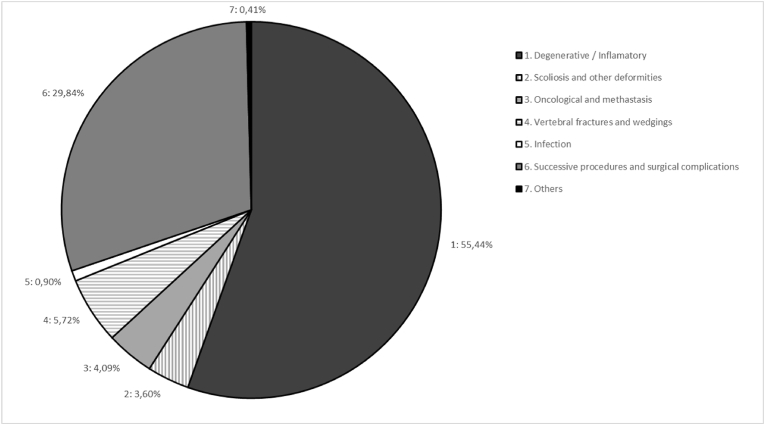
Coded diagnosis frequency Pie-chart indicating the frequency of coded diagnosis in the studied population.

**Fig. 2:**
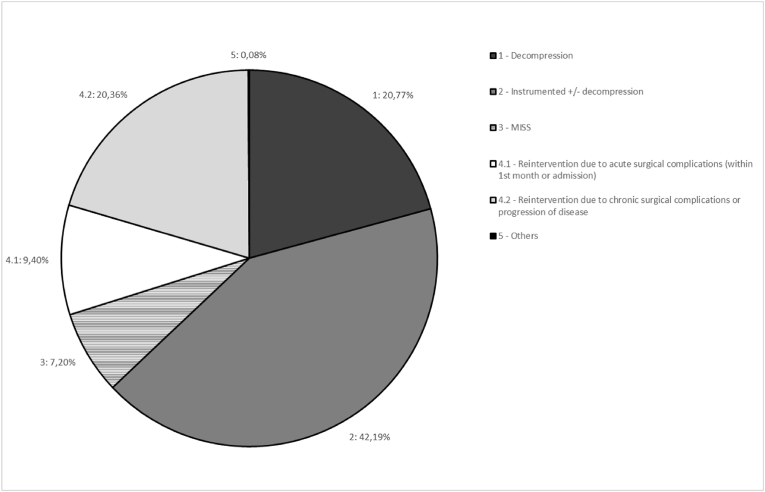
Coded treatment frequency Pie-chart indicating the frequency of coded treatments in the studied population.

A total of 1336 surgical procedures performed in patients over 13 years of age by the Spinal Unit at our center between April 2018 and April 2023 were potentially eligible for statistical analysis. Further details of this Unit can be consulted in a recent related paper (Rodríguez et al., 2023) and characteristics of patients' population are resumed through the analysed variables in Table 1. Exclusion criteria involved 124 cases: procedures consisting of radiofrequency neurolysis, local nerve infiltrations and spinal cord stimulator implantations (pain treatment percutaneous procedures), and those procedures performed by the Unit on nerve plexus or peripheral nerves were excluded from the analysis as well (Susanibar et al., 2025). This subset-excluded group also comprised 11 cases of non-specific diagnosis or treatments that were subsequently and properly codified (Fig. 1, Fig. 2). Therefore, we analysed a total of 1223 procedures performed on 1059 patients (124 patients were operated on twice, 27 patients three times, 10 patients four times and three patients were operated on five times in this period of five years) (see Table 2).

**Table 1:** Study population characteristics.

Variables	
**Age** (years), median (IQR)	62 (51; 72)
**BMI** (Kg/m^2^), median (IQR)	27.7 (24.6; 30.8)
**Preoperative,** median (IQR)	
Hb (g/dL)	13.9 (12.7; 15.1)
PT (secs)	11 (10.1; 11)
aPTT (secs)	27 (25.6; 29)
Platelets (10^3^/mm^3^)	253 (211; 301)
Haematological conditions or AP/OAC treatments, n (%)	181 (17)
**Region,** n (%)	
Cervical	158 (12.9)
Dorsal	65 (5.3)
Lumbar	809 (66.1)
Sacroccigeal	10 (0.8)
Others (pelvic and occipital)	5 (0.4)
Combined regions	176 (14.4)
**Position,** n (%)	
Prone	941 (77.1)
Supine	125 (10.2)
Lateral	15 (1.2)
Genupectoral	122 (10)
Combined positions	18 (1.5)
**Procedures,** n (%)	
Instrumented	757 (61.9)
MISS	141 (11.5)
Deformity	93 (7.6)
Succesive/Re-Do	401 (32.8)
≥3 levels	277 (22.6)
**TXA,** n (%)	635 (51.9)
**Intraoperative EBL** (cc), n (%)	
<100 cc	774 (63.3)
100-500 cc	238 (19.5)
≥500 cc	47 (3.8)
**Intraoperative transfusion,** n (%)	
RBC	75 (6.1)
Plasma	6 (0.5)
Platelets	1 (0.1)
**Postoperative Hb** (g/dl), mean (SD)	13.09 (2)
**Postoperative RBC transfusion,** n (%)	116 (9.5)
**Days of admission**, median (IQR)	5 (3; 11)

Abbreviations: Hb = hemoglobin; PT = prothrombin time; aPTT = activated partial thromboplastin time; AP/OAC = antiplatelet/oral anticoagulation treatments; MISS = minimally invasive spinal surgery; TXA = tranexamic acid; EBL = estimated blood loss; RBC = red blood cells; IQR = interquartile range; SD = standard deviation.

**Table 2:** Unweighted and weighted patients characteristics according to tranexamic acid (TXA) use.

	Unweighted			Weighted		
	No Tranexamic Acid (n = 580)	Tranexamic Acid (n = 632)	p-value	No Tranexamic Acid	Tranexamic Acid	p-value
**CLINICAL DATA**						
**Coded diagnosis, n (%)**						
1 - Degenerative/Inflammatory	377 (65.0%)	299 (47.3%)	<0.001	289.4 (46.3%)	298.0 (47.2%)	0.358
2 – HBR diagnoses^a^	39 (6.7%)	88 (13.9%)		83.9 (13.4%)	88.0 (13.9%)	
3 - Other deformities, other tumours, osteoporotic wedging fractures	17 (2.9%)	19 (3.0%)		21.6 (3.5%)	19.0 (3.0%)	
4 - Infection	2 (0.3%)	9 (1.4%)		3.1 (0.5%)	9.0 (1.4%)	
5.1 - Acute post-surgical complications (at 1 month)	63 (10.9%)	46 (7.3%)		79.4 (12.7%)	46.0 (7.3%)	
5.2 - Chronic or progressive complications of the disease	82 (14.1%)	171 (27.1%)		148.2 (23.7%)	171.0 (27.1%)	
**Coded treatment, n (%)**						
1 – Decompression	172 (29.7%)	78 (12.3%)	<0.001	82.8 (13.2%)	78.0 (12.4%)	0.443
2 – Instrumented	204 (35.2%)	311 (49.2%)		277.2 (44.3%)	310.0 (49.1%)	
3 – MISS	61 (10.5%)	25 (4.0%)		27.0 (4.3%)	25.0 (4.0%)	
4.1 – Acute reinterventions (<1 month)	66 (11.4%)	49 (7.8%)		74.7 (12.0%)	49.0 (7.8%)	
4.2 -Non-acute reintervention (>1 month)	77 (13.3%)	169 (26.7%)		163.6 (26.2%)	169.0 (26.8%)	
**Region, n (%)**						
1 - Cervical	102 (17.7%)	57 (9.0%)	<0.001	53.5 (8.6%)	57.0 (9.0%)	0.658
2 - Dorsal	25 (4.3%)	39 (6.2%)		40.3 (6.5%)	39.0 (6.2%)	
3 - Lumbar	396 (68.6%)	403 (63.9%)		425.7 (68.2%)	402.0 (63.8%)	
4 - Others	7 (1.2%)	3 (0.5%)		2.8 (0.4%)	3.0 (0.5%)	
5 – Combined	47 (8.1%)	129 (20.4%)		101.7 (16.3%)	129.0 (20.5%)	
**Age, median [IQR]**	60.0 [48.0; 70.5]	64.0 [52.0; 72.3]	<0.001	65.0 [55.0; 72.0]	64.0 [52.0; 72.0]	0.903
**BMI, median [IQR]**	27.5 [24.3; 30.1]	27.8 [24.8; 31.2]	0.024	28.1 [25.1; 31.2]	27.8 [24.8; 31.2]	0.597
**PREOPERATIVE DATA**						
**Haemoglobin, median [IQR]**	14.1 [12.8; 15.3]	13.8 [12.7; 14.9]	0.003	14.0 [12.6; 14.9]	13.8 [12.7; 14.9]	0.793
**Prothrombin time (seconds), median [IQR]**	10.9 [10.0; 11.0]	11.0 [10.2; 11.1]	0.175	11.0 [10.0; 11.3]	11.0 [10.2; 11.1]	0.647
**aPTT (seconds), median [IQR]**	27.0 [25.6; 29.0]	27.1 [25.6; 29.3]	0.507	27.9 [26.0; 29.9]	27.1 [25.6; 29.3]	0.613
**Platelets (10^3^/mm^3^), median [IQR]**	251 [210; 303]	255 [213; 300]	0.883	251 [197; 292]	255 [214; 299]	0.297
**Antiplatelet agents/Anticoagulants n (%)**	115 (19.9%)	121 (19.3%)	0.783	141 (22.7%)	120 (19.1%)	0.332
**INTRAOPERATIVE DATA**						
**Instrumented**	254 (43.9%)	498 (79.0%)	<0.001	487.8 (78.2%)	197.0 (79.0%)	0.764
**MISS**	104 (18.0%)	35 (5.6%)	<0.001	36.3 (5.8%)	35.0 (5.6%)	0.894
**Deformity**	14 (2.4%)	78 (12.4%)	<0.001	61.5 (9.9%)	78.0 (12.4%)	0.529
**Reoperation**	162 (28.0%)	233 (37.0%)	0.001	254.5 (40.9%)	233.0 (37.0%)	0.401
**≥3 levels**	72 (12.4%)	201 (31.9%)	<0.001	179.6 (28.8%)	201.0 (32.0%)	0.492
**Patient Position**						
1 – Prone position	391 (67.5%)	541 (85.9%)	<0.001	535.0 (85.7%)	540.0 (85.9%)	0.044
2 - Supine position	82 (14.2%)	41 (6.5%)		46.4 (7.4%)	41.0 (6.5%)	
3 – Lateral decubitus	8 (1.4%)	6 (1.0%)		13.1 (2.1%)	6.0 (1.0%)	
4 - Prone genupectoral	94 (16.2%)	28 (4.4%)		27.7 (4.4%)	28.0 (4.5%)	
5 – More than one position	4 (0.7%)	14 (2.2%)		1.9 (0.3%)	14.0 (2.2%)	
**Intraoperative bleeding volume (mL) (Estimated Blood Loss)**	337.1 (387.0)	513.6 (480.4)	0.001			
<500 cc	75 (12.9%)	212 (33.5%)	<0.001	232.6 (37.2%)	212.0 (33.6%)	0.693
≥500 cc	18 (3.1%)	146 (23.1%)		128.4 (20.5%)	145.0 (23.0%)	
**Intraop Transfusion** (Red blood cells)	16 (2.8%)	59 (9.3%)	<0.001	62.4 (10.0%)	59.0 (9.4%)	0.863
**Intraop Transfusion Volume (Units)**	0.1 (0.8)	0.2 (0.7)	0.020	0.2 (0.05)	0.2 (0.03)	0.869
**median [IQR]**	0.0 [0.0; 0.0]	0.0 [0.0; 0.0]	<0.001	0.0 [0.0; 0.0]	0.0 [0.0; 0.0]	0.962
**POST-OPERATIVE DATA**						
**Postop transfusion**	28 (4.8%)	87 (13.8%)	<0.001	90.2 (14.4%)	87.0 (13.8%)	0.863
**Postop transfusion Volume (Units)**	0.2 (1.4)	0.6 (2.8)	0.002	0.5 (0.19)	0.6 (0.11)	0.718
**median [IQR]**	0.0 [0.0; 0.0]	0.0 [0.0; 0.0]	<0.001	0.0 [0.0; 0.0]	0.0 [0.0; 0.0]	0.883
**Postop haemoglobin - 24 h (estimation bleeding)**	11.5 (2.1)	10.9 (2.0)	<0.001	10.5 (0.16)	10.9 (0.09)	0.039
Post haemoglobin <8 (Bleeding)	9 (3.0%)	33 (6.6%)	0.026	24.0 (5.4%)	33.0 (6.7%)	0.110
**COMPLICATIONS**						
**Days of Admission, median [IQR]**	3.0 [2.0; 8.0]	7.0 [4.0; 14.0]	<0.001	5.0 [3.0; 11.0]	7.0 [4.0; 14.0]	0.004
**Infection**	51 (8.9%)	93 (14.9%)	0.001	93.7 (15.2%)	93.0 (14.9%)	0.920
**Death**	21 (3.7%)	18 (2.9%)	0.444	61.0 (9.9%)	18.0 (2.9%)	0.002
**Other General Complications**	104 (18.1%)	199 (31.7%)	<0.001	164.2 (26.7%)	198.0 (31.6%)	0.256
**Bleeding estimation**						
**Haemoglobin change, mean (SD)**	−2.1 (1.7)	−2.7 (1.9)	<0.001	−2.8 (0.19)	−2.7 (0.09)	0.481
**Decrease ≥ 4 (bleeding), n (%)**	30 (10.1%)	101 (20.2%)	<0.001	95.5 (21.4%)	101.0 (20.3%)	0.825

Abbreviations: HBR = high bleeding risk; MISS = minimally invasive spinal surgery; BMI = body mass index; aPTT = activated partial thromboplastin time; IQR = interquartile range; SD = standard deviation.

a Metastatic tumour, complex trauma, or scoliosis.

**Table 3:** Baseline characteristics of transfuded and non-transfused groups adjusting by propensity score weighting.

	No transfusion	Transfusion	p-value
**CLINICAL DATA**			
**Coded diagnosis,** n (%)			
1 - Degenerative/Inflammatory	559.1 (54.7%)	28.3 (12.0%)	<0.001
2 – HBR diagnoses^a^	115.5 (11.3%)	56.4 (24.0%)	
3 - other deformities, other tumours, osteoporotic wedging fractures	37.6 (3.7%)	3.0 (1.3%)	
4 - Infection	2.6 (0.3%)	9.4 (4.0%)	
5.1 - Acute post-surgical complications (at 1 month)	55.1 (5.4%)	70.3 (29.9%)	
5.2 - Chronic or progressive complications of the disease	251.7 (24.6%)	67.4 (28.7%)	
**Coded treatment, n (%)**			
1 – Decompression	152.1 (14.9%)	8.8 (3.7%)	<0.001
2 – Instrumented	500.0 (48.9%)	87.2 (37.2%)	
3 – MISS	52.0 (5.1%)	0.0 (0.0%)	
4.1 – Acute reinterventions (<1 month)	52.5 (5.1%)	71.3 (30.4%)	
4.2 -Non-acute reintervention (>1 month)	265.1 (25.9%)	67.5 (28.7%)	
**Region, n (%)**			
1 - Cervical	103.5 (10.2%)	7.0 (3.0%)	<0.001
2 - Dorsal	59.7 (5.9%)	19.6 (8.4%)	
3 - Lumbar	723.3 (71.0%)	104.4 (44.5%)	
4 - Others	5.8 (0.6%)	0.0 (0.0%)	
5 – Combined	127.0 (12.5%)	103.7 (44.2%)	
**Age, median [IQR]**	62.0 [52.0; 72.0]	68.0 [57.0; 74.3]	0.002
**BMI, median [IQR]**	28.1 [24.9; 31.2]	27.0 [25.0; 30.2]	0.261
**PREOPERATIVE DATA**			
**Haemoglobin, median [IQR]**	14.1 [13.1; 15.0]	12.2 [10.0; 13.6]	<0.001
**PT, median [IQR]**	11.0 [10.0; 11.0]	11.0 [10.1; 12.0]	0.094
**aPTT, median [IQR]**	27.0 [25.8; 29.0]	28.1 [26.0; 32.7]	0.065
**Platelets, median [IQR]**	256 [214; 294]	232 [175; 296]	0.083
**Antiplatelet agents/Anticoagulants, n (%)**	193.9 (19.0%)	67.0 (29.1%)	0.088
**INTRAOPERATIVE DATA**			
**Instrumented, n (%)**	771.0 (75.7%)	213.7 (91.1%)	0.001
**MISS, n (%)**	69.3 (6.8%)	2.0 (0.9%)	<0.001
**Deformity, n (%)**	77.6 (7.6%)	61.9 (26.4%)	<0.001
**Reoperation, n (%)**	333.9 (32.8%)	153.6 (65.4%)	<0.001
**≥ 3 levels, n (%)**	236.0 (23.2%)	144.6 (61.6%)	<0.001
**Patient Position, n (%)**			
1 – Prone position	854.0 (83.9%)	221.0 (94.2%)	<0.001
2 - Supine position	80.4 (7.9%)	7.0 (3.0%)	
3 – Lateral decubitus	18.1 (1.8%)	1.0 (0.4%)	
4 - Prone genupectoral	54.7 (5.4%)	1.0 (0.4%)	
5 – More than one position	11.3 (1.1%)	4.7 (2.0%)	
**EBL (cc)**			
NA	468.6 (45.9%)	69.9 (29.8%)	<0.001
< 500	406.3 (39.8%)	38.3 (16.3%)	
≥ 500	146.8 (14.4%)	126.6 (53.9%)	
**POSTOPERATIVE DATA**			
**Postop haemoglobin 48h**	11.3 (0.09)	8.9 (0.14)	<0.001
Postop haemoglobin <8 (Bleeding)	7.0 (1.0%)	50.0 (21.7%)	<0.001
**COMPLICATIONS**			
**Days of Admission, median [IQR]**	5.0 [3.0; 9.0]	23.0 [8.0; 40.0]	<0.001
**Infection, n (%)**	91.8 (9.1%)	94.9 (40.6%)	<0.001
**Death, n (%)**	28.4 (2.8%)	50.5 (21.7%)	<0.001
**Other General Complications, n (%)**	217.2 (21.5%)	145.0 (62.0%)	<0.001
**Bleeding estimation**			
**Haemoglobin change, mean (SD)**	−2.6 (0.08)	−3.0 (0.31)	0.276
**median [IQR]**	−2.5 [-3.5; −1.7]	−2.9 [-4.7; −1.2]	0.341
Decrease ≥4 (bleeding), n (%)	110.2 (15.5%)	86.3 (37.4%)	<0.001

Abbreviations: HBR = high bleeding risk; MISS = minimally invasive spinal surgery; BMI = body mass index; aPTT = activated partial thromboplastin time; PT = prothrombin time; EBL = estimated blood loss; IQR = interquartile range; SD = standard deviation.

a Metastatic tumour, complex trauma, or scoliosis.

**Table 4:** Baseline characteristics by mortality adjusting by propensity score weighting.

	No death	Death	p-value
**CLINICAL DATA**			
**Coded diagnosis, n (%)**			
1 - Degenerative/Inflammatory	580.8 (50.1%)	1.3 (1.7%)	<0.001
2 – HBR diagnoses^a^	120.6 (10.4%)	42.4 (53.7%)	
3 - other deformities, other tumours, osteoporotic wedging fractures	37.0 (3.2%)	3.6 (4.5%)	
4 - Infection	9.6 (0.8%)	2.4 (3.1%)	
5.1 - Acute post-surgical complications (at 1 month)	104.5 (9.0%)	19.8 (25.1%)	
5.2 - Chronic or progressive complications of the disease	307.1 (26.5%)	9.4 (12.0%)	
**Coded treatment, n (%)**			
1 – Decompression	151.3 (13.0%)	7.4 (9.3%)	0.198
2 – Instrumented	541.2 (46.7%)	35.1 (44.5%)	
3 – MISS	46.3 (4.0%)	4.8 (6.1%)	
4.1 – Acute reinterventions (<1 month)	102.9 (8.9%)	19.8 (25.1%)	
4.2 -Non-acute reintervention (>1 month)	318.1 (27.4%)	11.9 (15.0%)	
**Region, n (%)**			
1 - Cervical	107.2 (9.3%)	2.3 (2.9%)	0.001
2 - Dorsal	57.4 (5.0%)	22.0 (27.8%)	
3 - Lumbar	774.3 (66.9%)	41.0 (52.0%)	
4 - Others	5.8 (0.5%)	0.0 (0.0%)	
5 – Combined	212.6 (18.4%)	13.7 (17.3%)	
**Age, median [IQR]**	64.0 [53.0; 72.0]	66.0 [56.0; 76.0]	0.499
**BMI, median [IQR]**	28.3 [25.1; 31.2]	25.7 [24.2; 27.6]	0.022
**PREOPERATIVE DATA**			
**Haemoglobin, median [IQR]**	13.9 [12.7; 14.9]	12.1 [11.3; 14.7]	0.044
**PT (seconds),median [IQR]**	11.0 [10.0; 11.0]	11.9 [10.8; 12.9]	0.005
**aPTT (seconds), median [IQR]**	27.0 [25.9; 29.0]	32.9 [26.2; 34.0]	0.113
**Platelets, median [IQR]**	253 [211; 294]	196 [174; 253]	0.010
**Antiplatelet agents/Anticoagulants, n (%)**	225.1 (19.5%)	34.1 (43.8%)	0.041
**INTRAOPERATIVE DATA**			
**Instrumented, n (%)**	901.7 (77.9%)	71.1 (90.1%)	0.096
**MISS, n (%)**	64.5 (5.6%)	3.9 (4.9%)	0.856
**Deformity, n (%)**	125.1 (10.8%)	6.5 (8.3%)	0.644
**Reoperation, n (%)**	451.2 (39.0%)	31.7 (40.1%)	0.940
**≥ 3 levels, n (%)**	340.1 (29.4%)	37.4 (47.4%)	0.165
**Patient Position, n (%)**			
1 – Prone position	990.7 (85.6%)	74.7 (94.6%)	0.167
2 - Supine position	84.8 (7.3%)	2.6 (3.3%)	
3 – Lateral decubitus	12.1 (1.0%)	0.0 (0.0%)	
4 - Prone genupectoral	55.6 (4.8%)	0.0 (0.0%)	
5 – More than one position	14.3 (1.2%)	1.7 (2.1%)	
**Bleeding volume (cc)**	394.0 (302.5)	804.8 (524.6)	<0.001
Negligible	514.7 (44.4%)	20.8 (26.3%)	0.001
< 500	417.5 (36.0%)	14.4 (18.2%)	
≥ 500	227.6 (19.6%)	43.8 (55.5%)	
**Intraop Transfusion**			
1 – Red blood cells	79.0 (6.8%)	41.3 (52.4%)	<0.001
**Intraop Transfusion Volume (Units)**	457.0 (25.80)	1088.8 (197.13)	0.002
**POST-OPERATIVE DATA**			
**Postop Transfusion**	141.9 (12.2%)	33.3 (42.2%)	0.003
**Postop Transfusion Volume (Units)**	0.5 (0.12)	0.9 (0.37)	0.315
**Postop haemoglobin 24h**			
Postop haemoglobin <8 (Bleeding)	49.8 (5.8%)	5.2 (7.1%)	0.749
**COMPLICATIONS**			
**Days of Admission, median [IQR]**	5.0 [4.0; 12.0]	15.0 [8.5; 30.4]	0.147
**Infection, n (%)**	175.2 (15.2%)	9.9 (12.5%)	0.668
**Other General Complications, n (%)**	313.7 (27.1%)	47.5 (60.2%)	0.005
**Bleeding estimation**			
**Haemoglobin change, mean (SD)**	−2.7 (0.09)	−3.0 (0.80)	0.754
**median [IQR]**	−2.6 [-3.7; −1.6]	−2.4 [-3.8; −1.3]	0.981
Decrease ≥4 (bleeding), n (%)	176.7 (20.6%)	18.8 (25.6%)	0.740

Abbreviations: HBR = high bleeding risk; BMI = body mass index; PT = prothrombin time; aPTT = activated partial thromboplastin time; MISS = minimally invasive spinal surgery; EBL = estimated blood loss; IQR = interquartile range; SD = standard deviation.

a Metastatic tumour, complex trauma, or scoliosis.

**Table 5:** Odds Ratio (OR) for transfusion estimated using generalized estimating equation (GEE) models.

	OR	95% IC OR	p-value
**Constant**	0.00		<0.001
**Coded diagnosis, n (%)**			
1 - Degenerative/Inflammatory	Ref.		
2 - HBR diagnoses^a^	3.03	0.97; 9.82	0.059
3 - other deformities, other tumours, osteoporotic wedging fractures	1.77	0.09; 13.40	0.632
4 - Infection	23.30	1.37; 1394	0.043
5.1 - Acute post-surgical complications (at 1 month)	10.70	3.39; 35.70	<0.001
5.2 - Chronic or progressive complications of the disease	2.02	0.75; 5.60	0.167
**aPTT (s)**			
< 25	Ref.		
25 – 30	1.35	0.53; 3.61	0.540
>30	5.92	1.98; 19.20	0.002
**≥ 3 levels**			
No	Ref.		
Yes	3.80	1.74; 8.61	0.001
**EBL (cc)**			
Negligible	Ref.		
<500 cc	1.09	0.41; 2.95	0.861
≥500 cc	9.94	4.02; 27.00	<0.001
**Days of admission**	1.03	1.01; 1.05	<0.001
**Other general complications**			
No	Ref.		
Yes	3.41	1.56; 7.57	0.002

Abbreviations: HBR = high bleeding risk; aPTT = activated partial thromboplastin time; EBL = estimated blood loss.

a Metastatic tumour, complex trauma, or scoliosis.

**Table 6:** Odds Ratio (OR) for mortality estimated using generalized estimating equation (GEE) models.

	OR	95% IC OR	p-value
**Constant**	0.04		<0.001
**TXA**			
No	Ref.		
Yes	0.25	0.1; 0.54	0.001
**aPTT (s)**			
< 25	Ref.		
25 – 30	0.37	0.1; 1.04	0.057
>30	3.67	1.6; 9.43	0.005
**EBL (cc)**			
Negligible	Ref.		
<500 cc	1.05	0.4; 2.87	0.931
≥500 cc	7.13	3.1; 17.80	<0.001
**Days of admission**	1.02	1.0; 1.03	0.001

Abbreviations: TXA = Tranexamic acid; aPTT = activated partial thromboplastin time; EBL = estimated blood loss.

181 patients of our series (17%) had significant past medical history related to haematological disorders, being on antiplatelet (AP) medication the most frequent one (10,8%) and mainly (1,6%) due to ischaemic heart disease or peripheral vasculopathy. Thirteen patients in our population had anaemia, being this preop condition defined as Hb values < 13 g/dL in males and <12 g/dL in females and treated with erythropoietin (EPO), ferrous sulphate or cyanocobalamin when required during the anaesthetic prehabilitation, enhancing patient's functional capacity. In addition to these pre-existing haematological conditions and clotting disorders, or the AP, AC and procoagulant medication use, the rest of preop analysed variables included age, body mass index (BMI) according to standardised categories proposed by the world health organisation (WHO), and parameters from blood tests as Hb, prothrombin time (PT), activated partial thromboplastin time (aPTT) and platelet count. Particular attention was paid when the preop codified diagnosis was considered part of the items included in National Health Service (NHS) definition of CSS (NHS England, 2021) such as deformity, spinal metastases or complex fractures. The two Jehova's Witness patients of our serie were registered as well (Gohel et al., 2005).

Considered intraop variables were spinal region/s involved in the procedure, patient's surgical position, codified treatment (again specifying those CSS cases), instrumented procedure, MISS, three or more spinal levels approached, and reinterventions. When not contraindicated, TXA was administered (Colomina et al., 2017) as an IV infusion of 10 mg kg−1 (Amchafibrin® 500 mg; Rottapharm S.L., 5-mL vials), 20 min before the surgical incision, followed by continuous perfusion of 2 mg kg h−1 up to surgical wound closure at completion of surgery. EBL during the surgical procedure was registered according to Anesthetists notes taking away the volume of fluids used during surgical wash-out from the total volume in suction's collector at the end of the operation, and it was codified for the statistical analysis in non-significant, 100-500 and > 500 cc groups. Volume and type of transfused products were also recorded intraop variables, and intraop cell-saver use was also specified across the database. Red blood cells (RBC) and plasma concentrates are 285 cc per administered unit in our Institution (coming from a single donor) and platelets concentrates have a 300 cc volume per unit, these last ones being obtained from four donors for each concentrate. We did not analyse the duration of surgical procedures.

The difference between preop Hb and Hb value 24 h after surgery was also considered for EBL purposes (with postop-Hb absolute values < 8, or differences with preop-Hb value > 4 points being used for the categorised groups in the statistical analysis). The rest of postop admission recorded variables were volume collected through surgical drains, volume and type of postop transfused products when required, number of days of admission and complications related to the surgical procedure, with particular consideration of readmissions and deaths during first month after surgery (mortality rate = 0.008). Postop infections (72 patients with confirmed microorganism, with 56 of them having a surgical site infection, 5.3%) or cases returning to theatre for revision surgery within a year after the original procedure (NHS England, 2021) were recorded as well. Postop haematoma was identified in 31 patients, requiring eight of them (0.7%) surgical evacuation. We did not analyse economic costs during postop admission. Open Evidence Artificial Intelligence (AI) was just used for the bibliographic search of the manuscript.

To account for treatment selection bias, propensity scores were estimated using a logistic regression model. The resulting c-statistic was 0-756, indicating good discriminative ability. Standardised morbidity ratio (SMR) weighting (Brookhart et al., 2013) was applied by assigning a weight of one to patients in the TXA group, while patients in the non-TXA group were weighted by the ratio of estimated propensity score to one minus the estimated propensity score.

To compare treatment groups, a complex survey design framework was applied, incorporating inverse probability of treatment weighting (IPTW) to adjust for potential confounding. Continuous variables were summarized using medians and interquartile ranges due to non-normal distributions. Group comparisons were performed using nonparametric methods appropriate for weighted data, such as Rao-Scott corrected rank tests or permutation-based approaches, as appropriate.

To estimate the association between treatment and the outcomes (transfusion and death), generalized estimating equations (GEE) were used, incorporating IPTW to account for the weighted structure of the data and to obtain robust standard errors.

All statistical analysis of data were performed using R software (version 4.2.3).

## 3 Results

In the unweighted analysis, patients receiving TXA had a significantly higher prevalence of HBRD diagnoses (13.9% vs. 6.7%; p < 0.001) and spinal deformity (12.4% vs. 2.4%; p < 0.001). Surgical procedures involving more than three levels were more frequent in the TXA group (31.9% vs. 12.4%; p < 0.001), as were reoperations (37.0% vs. 28.0%; p = 0.001). A prone position was more commonly used in patients who received TXA (85.9% vs. 67.5%; p < 0.001). TXA administration was associated with a significantly higher estimated intraoperative blood loss (513.6 mL vs. 337.1 mL; p = 0.001) and an increased rate of intraoperative red blood cell transfusions (9.3% vs. 2.8%; p < 0.001). These patients also received a greater postoperative transfusion volume (0.6 vs. 0.2 units; p = 0.002) and showed a larger drop in hemoglobin levels postoperatively (10.9 vs. 11.5 g/dL; p < 0.001). Additionally, the TXA group experienced higher rates of overall complications (31.7% vs. 18.1%; p < 0.001), infections (14.9% vs. 8.9%; p = 0.001), and longer hospital stays (median 7 vs. 3 days; p < 0.001).

## 4 Discussion

### 4.1 Key findings

Stratifying a population for the administration of a drug means identifying the best subgroups for whom it may be safe and effective. In our series, no epileptic seizures nor impairment in renal function were observed in the TXA group, and only three ischaemic events were seen across the 1223 surgical interventions during the first postoperative month, two of them in patients with previous stroke or ischaemic heart disease, which are formal contraindications for TXA administration (Gong et al., 2019).

Updated analysis confirmed that TXA was more frequently used in higher-risk scenarios: patients receiving TXA had a significantly higher prevalence of HBRD diagnoses (13.9% vs. 6.7%; p < 0.001), spinal deformities (12.4% vs. 2.4%; p < 0.001), surgeries involving more than three levels (31.9% vs. 12.4%; p < 0.001), and reoperations (37.0% vs. 28.0%; p = 0.001). Prone positioning was more commonly used in the TXA group (85.9% vs. 67.5%; p < 0.001).

A general consensus accepts the use of TXA in spinal surgery in elderly, obese patients or those with preoperative Hb < 13 g/dL. Its use is also well supported in instrumented, non-MISS thoracolumbar surgeries involving >3 levels, especially in re-do procedures and in those with expected bleeding >1000 cc (Colomina et al., 2017). In line with this, TXA administration in our series was strongly associated with estimated intraoperative blood loss (EBL): patients receiving TXA had higher EBL (513.6 mL vs. 337.1 mL; p = 0.001), greater intraoperative transfusion rates (9.3% vs. 2.8%; p < 0.001), larger postoperative Hb (10.9 vs. 11.5 g/dL; p < 0.001), and higher postoperative transfusion volume (0.6 vs. 0.2 units; p = 0.002).

According to the literature, high EBL is expected in diagnoses such as scoliosis, metastasis, and complex polytrauma fractures requiring spinal instrumentation (Chen et al., 2020; Garríguez et al., 2023; Martín-et al., 2024). The association between high EBL and low platelet counts or use of AP/OAC therapy is also well documented (Chow et al., 2021; Yu et al., 2024). In our series, EBL >500 cc was significantly more frequent in re-do, multilevel, and combined topographical spinal region procedures (p < 0.001) (Butler et al., 2011). A postoperative Hb drop ≥4 g/dL was significantly related to non-MISS procedures, highlighting the *benefits of minimally invasive approaches in reducing intraoperative bleeding*.

Easing venous return by reducing intra-abdominal pressure was also critical to reducing EBL, as demonstrated in patients positioned supine, lateral, or in genupectoral (“knees-to-chest”) positions (Willner et al., 2016). Conversely, prone positioning, though necessary in many posterior approaches, was associated with significantly higher transfusion rates.

Increased EBLs were significantly associated with the main indicators of unfavorable postoperative outcomes in our study: infection and prolonged hospital admission, both with p < 0.001. Literature supports the use of postoperative Hb < 8 g/dL (Pull ter et al., 2010; Nemani et al., 2020) and ΔHb ≥4 g/dL (Purvis et al., 2018) as estimators of intraoperative bleeding and predictors of adverse outcomes. In our multivariable analysis, ΔHb ≥4 g/dL was more strongly associated with worse overall postoperative evolution and a greater number of complications, while Hb < 8 g/dL was more significantly associated with acute postoperative events. The ΔHb ≥4 group had a significantly higher rate of reoperations during the first year post-surgery (p = 0.001).

Intraoperative and postoperative blood transfusions, required in 6.7% and 9.5% of patients respectively, were associated with significantly higher rates of postoperative infections, medical complications, longer hospital stays, and mortality (p < 0.001) (Kato et al., 2016; Falsetto et al., 2022; Janssen et al., 2015). Among transfused patients, 91.5% received allogeneic RBCs. Transfusions were significantly more frequent in patients with HBRD (13.9% vs. 6.7%; p < 0.001), procedures with high EBL, ΔHb ≥4 g/dL, and those under AP/OAC treatment.

According to the type of surgery, transfusions were more common in high-bleeding-risk surgeries (HBRS), and among the 141 MISS procedures, only one case required blood products. Prone position was associated with significantly more transfusions, whereas the genupectoral position may be preferable for simple lumbar decompressions, though it is not applicable for fixations due to flat-back syndrome risk.

Multivariable analysis confirmed that transfusion risk was independently associated with HBRD, diagnosis of acute postoperative complications (OR = 10.70; p < 0.001), aPTT >30 s (OR = 5.92; p = 0.002), procedures involving >3 levels (OR = 3.80; p = 0.001), EBL ≥500 cc (OR = 9.94; p < 0.001), prolonged hospital stay (OR = 1.03; p < 0.001), and occurrence of general complications (OR = 3.41; p = 0.002) (Basques et al., 2015).

Mortality was significantly related to preoperative HBRD (53.7% vs. 10.4%; p < 0.001), lower Hb, prolonged PT (p = 0.005), lower platelet counts (p = 0.010), and use of AP/OAC therapy (p = 0.041). Intraoperative blood loss was significantly higher among deceased patients (804.8 vs. 394.0 mL; p < 0.001), as was intraoperative transfusion rate (52.4% vs. 6.8%; p < 0.001) and transfused volume (p = 0.002). Postoperative transfusion was also significantly more frequent in this group (p = 0.003) (Peul and Moojen, 2016).

The physiological impact of postoperative anemia includes reduced tissue oxygenation in the context of heightened metabolic demands due to the surgical stress response (Meng et al., 2017). These patients showed significantly higher rates of infection, mortality, and longer hospitalizations.

Despite being used in more complex and higher-risk surgeries, TXA was independently associated with a reduced risk of mortality in the multivariable model (OR = 0.25; p = 0.001), mirroring findings from other clinical settings (Shakur et al., 2010).

Therefore, we reinforce our proposal for a protocol of IV *TXA administration in spinal surgery for patients >60 years old, with BMI >27, preoperative Hb <*13 g/dL, *or aPTT >27 s*. TXA should also be administered in cases of *spinal metastases, deformities, complex fractures, thoracic or lumbar non-MISS surgeries, and instrumented procedures involving >3 levels or re-do surgeries*. When two or more of these factors are present, TXA administration is recommended.

When not contraindicated, TXA should be administered as a 10 mg/kg IV infusion 20 min prior to incision, followed by continuous perfusion of 2 mg/kg/h until surgical wound closure.

### 4.2 Limitations

Our study was limited by its retrospective, single-centred and observational character, resulting in a lower strength of evidence. There was also a lack of analysis of the economic costs of TXA use in comparison to extended hospital admission related to major bleedings, nor did we analyse the duration of the surgical procedure or the length of the wound as variables potentially related to EBL. Further studies considering the aforementioned variables are needed in order to fully analyse the impact of the administration of TXA in patients undergoing spinal surgery. Multicenter, prospective, randomized clinical trials regarding this drug will achieve more robust evidence regarding its safety, benefits and indications in spinal surgery.

## 5 Conclusions

Our study demonstrates that TXA, when administered to carefully selected patients, significantly reduces mortality and adverse outcomes in spinal surgery without increasing thromboembolic or renal complications. The benefit is most evident in high-bleeding-risk scenarios, particularly in non-MISS thoracolumbar procedures, deformity corrections, complex fractures, multilevel instrumentation, and reoperations.

Based on these findings, we propose the implementation of a stratified TXA protocol that integrates both patient-related factors (age >60 years, BMI >27, preoperative anemia, coagulopathy, or anticoagulant/antiplatelet therapy) and procedure-related factors (non-MISS surgery, thoracic or lumbar approaches, deformity, metastases, instrumentation of >3 levels, or re-do cases).

Adopting this protocol can improve perioperative safety, reduce transfusion requirements, and ultimately enhance survival in high-risk spinal surgery populations. Future multicenter prospective trials are warranted to confirm and refine these recommendations.

## Statements and declarations

This research did not receive any specific grant from funding agencies in the public, commercial, or not-for-profit sectors.

## Declaration of competing interest

The authors declare that they have no known competing financial interests or personal relationships that could have appeared to influence the work reported in this paper.
